# Increasing breastfeeding initiation and duration in women with a body mass index ≥ 30 kg/m^2^: developing an intervention from theory to acceptability

**DOI:** 10.1186/s13006-025-00720-w

**Published:** 2025-04-10

**Authors:** Stephanie Lyons, Sinead Currie, Sarah Peters, Dame Tina Lavender, Emilie Turnbull, Ria Bhatia, Zaynab Khan, Debbie M. Smith

**Affiliations:** 1 Division of Psychology & Mental Health, School of Health Sciences, Faculty of Biology, Medicine & Health, University of Manchester, Manchester, UK; 2 Division of Psychology, Faculty of Natural Sciences, University of Stirling, Stirling, UK; 3 Centre for Childbirth, Women’s and Newborn Health, Liverpool School of Tropical Medicine, Liverpool, UK

**Keywords:** Breastfeeding, Body mass index, Intervention development, Acceptability, Psychological, Behaviour change

## Abstract

**Background:**

Breastfeeding has many health benefits for both mother and child, but rates of initiation and duration amongst women with a BMI ≥ 30 kg/m^2^ are low. Few interventions aiming to increase breastfeeding in this group have been successful; likely because existing interventions do not target psychological factors. Therefore, this study aimed to design and explore the acceptability of a newly developed psychological intervention to increase breastfeeding initiation and duration in women with a BMI ≥ 30 kg/m^2^.

**Methods:**

The Medical Research Council’s Complex Intervention Development Framework was followed to design the intervention. A ‘breastfeeding workbook’ was developed, based on literature and psychological theory, and filled with tailored information and activities. A Patient and Public Involvement group of women with a BMI ≥ 30 kg/m^2^ who had breastfed, health professionals and researchers were consulted throughout the process, selecting the intervention content, format and delivery methods. Thirteen women with a BMI ≥ 30 kg/m^2^ then reviewed the workbook and took part in in-depth qualitative interviews to assess its acceptability. Thematic analysis was conducted, informed by the Theoretical Framework of Acceptability version 2.

**Results:**

The findings reveal the intervention is acceptable to the target population; women believe the intervention shows promise for increasing breastfeeding initiation and duration, is representative of their experiences, is accessible, and aligns with their belief system. They valued that the intervention provided realistic expectations of breastfeeding, options to overcome the challenges of breastfeeding in the real-world and supported them to breastfeed without inducing stigma or shame regarding their weight or infant feeding practices. Suggestions for improvement are also included, such as incorporating audio and video content as alternatives to written text and translation options.

**Conclusions:**

An acceptable, psychological intervention was developed to increase breastfeeding initiation and duration in women with BMIs ≥ 30 kg/m^2^. These findings can inform maternity and breastfeeding care, future research directions and intervention development.

## Background

An increasing body of research acknowledges that women with a BMI ≥ 30 kg/m^2^ are less likely than those with lower BMIs to initiate and maintain breastfeeding [1, 2]. Physical and biological challenges, such as larger breasts and delayed lactogenesis II in women with higher BMIs can decrease the likelihood of breastfeeding success [1]. However, psychological factors (i.e. those that affect or arise in the mind, such as attitudes, knowledge, and beliefs) are also important for breastfeeding behaviours [1]; intending to breastfeed, breastfeeding knowledge, and belief in ability to breastfeed have been consistently linked to breastfeeding success within the general population [3–5]. However, women with a BMI ≥ 30 kg/m^2^ are less likely to intend, and have confidence in their ability, to breastfeed [6, 7], which could explain lower breastfeeding rates amongst this group. Furthermore, a systematic review reported women with a BMI ≥ 30 kg/m^2^ are also less likely believe in their breast milk’s nutritional adequacy and sufficiency, that the others around them prefer breastfeeding to other infant feeding methods, know others who have breastfed, and have poorer body image compared with women with lower BMIs [8].

Few breastfeeding interventions have been designed specifically for women with a BMI ≥ 30 kg/m^2^, despite the recognised challenges [1]. To date, only three have been identified [9], focusing on providing breastfeeding and/or nutritional education, and telephone support. Of those investigating effectiveness, only one has reported any increase in breastfeeding behaviour [10]; however, this sample had a higher maternal age than previous intervention studies, was highly educated, and intended to breastfeed prior to intervention delivery, all factors which are independently associated with higher likelihood of breastfeeding success [11–13]. Furthermore, a similar intervention delivered in areas of higher deprivation, where having a higher BMI is more likely, found no effect beyond 2 weeks [14]. Therefore, there remains a significant need to develop effective interventions to increase breastfeeding initiation and duration in women with BMIs ≥ 30 kg/m^2^.

The Medical Research Council (MRC) provides guidance for developing complex interventions [15]. The process begins with an evidence synthesis, identifying any gaps in the existing literature, and completing a systematic review if appropriate. Behaviour change theory should then be considered, to explain the mechanisms behind any changes that occur. Before assessing its effectiveness, it is recommended that researchers evaluate the intervention’s acceptability with the target population [15]; if an intervention is acceptable, it is more likely to be delivered and completed as intended [16].

The Theoretical Framework of Acceptability (TFAv2) [16] posits acceptability as a multi-faceted construct, with seven important components: affective attitude (how an individual feels about the intervention); burden (the perceived effort required to participate); ethicality (how the intervention fits with their value system); intervention coherence (how far the individual understands the intervention and how it works); opportunity costs (how far benefits, profits or values have to be given up to engage with the intervention); perceived effectiveness (how far it is believed to be likely to achieve its purpose); and self-efficacy (their confidence to perform the behaviours required to participate). Qualitative interviews allow in-depth exploration of the acceptability of a new intervention [17]. Several studies have successfully applied the TFAv2 to qualitative interview data to assess the acceptability of an intervention [18–21]. Therefore, this study aimed to assess the acceptability of a newly designed, psychological intervention to support breastfeeding in women with a BMI ≥ 30 kg/m^2^. The TFAv2 was utilised to inform the development and analysis of qualitative interviews, conducted after intervention delivery with pregnant and breastfeeding women with a BMI ≥ 30 kg/m^2^.

## Methods

**Fig. 1:**
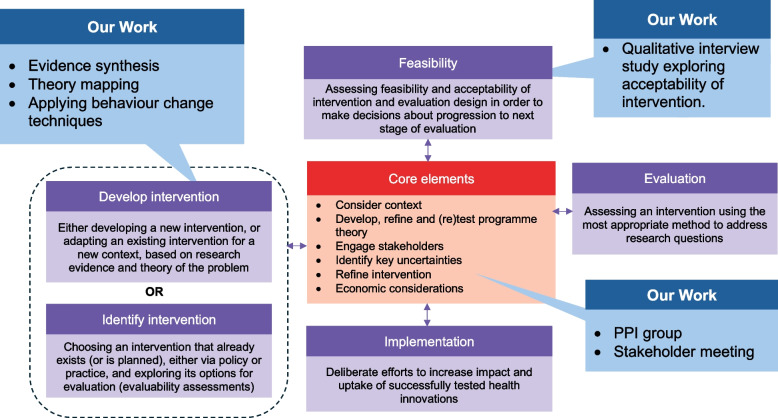
Key elements for developing and evaluating complex interventions (adapted from Skivington et al., 2021) [15]

The MRC complex intervention framework informed the development process of a psychological intervention to increase breastfeeding initiation and duration in women with a BMI ≥ 30 kg/m^2^. This paper has been reported in line with the COREQ checklist [22]. Ethical approval was given for the study from the University Ethics Committee (Ref: 17,963). The design process is described in detail below and summarised in Fig. 1.

### Intervention design

Three steps were conducted to design the intervention:

i. *Synthesising the evidence*
  Existing breastfeeding interventions for women with a BMI ≥ 30 kg/m^2^ were first examined. Research investigating the association between psychological factors and breastfeeding in women with a BMI ≥ 30 kg/m^2^ was identified, and a quantitative systematic review was conducted [8]. From this review, a framework of psychological factors was developed; this was then used to develop a list of codes, to deductively analyse qualitative interview data and explore whether these factors were part of women’s lived experiences of breastfeeding [23]. A meta-synthesis of the qualitative literature exploring the perceptions and experiences of women with a BMI ≥ 30 kg/m^2^ who breastfeed was also conducted [24]. The findings from these studies were presented to breastfeeding women with a BMI ≥ 30 kg/m^2^, health professionals and researchers in a stakeholder meeting; participants then engaged in a series of activities to select and prioritise the intervention content (psychological factors), format and delivery methods.
ii. *Theory mapping*
  Whilst the MRC framework guided the steps to intervention development, behaviour change theory was required to predict and explain any changes in breastfeeding behaviour as a result of the intervention [15]. Following the stakeholder meeting, a five-step systematic process of theory identification was conducted. The evidence synthesis was utilised to determine the target theoretical constructs (step 1), and essential and desirable eligibility criteria were developed for suitable theories (step 2). A comprehensive list of theories, using the *ABC of Behaviour Change Theories* [25] was then collated (step 3) and systematically considered in turn against the pre-determined criteria (step 4). Those which were deemed appropriate upon first consideration were examined in more detail (step 5). This resulted in the selection of the I-Change Model [26] to the intervention, to explain the likely process of change.
iii. *Applying behaviour change techniques*
  Once the intervention content and theory had been set, as for theory-mapping, the five-step systematic process was conducted to identify appropriate behaviour change techniques. The evidence synthesis was utilised to determine the target theoretical constructs (step 1), and essential and desirable eligibility criteria were developed for suitable techniques (step 2). A comprehensive list of techniques, using the Behaviour Change Technique Taxonomy v1 [27] was then collated (step 3) and systematically considered in turn against the pre-determined criteria for each of the 10 selected psychological factors (step 4). Those which were deemed appropriate upon first consideration were examined in more detail (step 5). This resulted in the selection of 17 techniques identified under Intervention Design. Those included were transformed into information or activities appropriate for the selected intervention format and delivery methods.

The Breastfeeding Workbook was co-produced with a Patient and Public Involvement (PPI) group of women with a BMI ≥ 30 kg/m^2^ who had breastfed, health professionals and researchers. It is informed by the I-Change Model [26], a psychological theory which states behaviours are determined by motivation and intentions (see above Theory mapping section). The intervention targets 10 important psychological factors for breastfeeding in women with higher BMIs [6, 8, 27]: planned infant feeding method and duration; general attitudes and beliefs about breastfeeding; belief in breast milk’s nutritional adequacy and sufficiency; belief about others’ infant feeding preferences; social, and factual knowledge; and confidence in ability to breastfeed, to breastfeed in social situations and to seek support. It also employs 17 behaviour change techniques [27] to help women to plan, initiate and maintain breastfeeding: Setting/reviewing goals (1.1, 1.5); Social support (3.1); Instruction on how to perform (4.1), and demonstration of the behaviour (6.1); Information on health (5.1) and social/environmental consequences (5.3); Social comparison (6.2); Information about others’ approval (6.3); Credible source (9.1); Pros and cons (9.2); Adding objects to (12.5) and restructuring the physical (12.1) and social environment (12.2); Verbal persuasion about capability (15.1); Mental rehearsal of successful performance (15.2); and Self-talk (15.4). The content is grouped into four sections: *why breastfeeding is important*, including myth-busting information, a pros and cons exercise and goal setting; *getting started,* including information about expressing, positioning and accessing support; *when you’re out and about,* including information and exercises to build confidence for social situations; and *when things get tough,* including information and exercises on overcoming challenges (complications, pain and returning to work). It is intended that the intervention be delivered in pregnancy and be worked through depending on user preference, either in one sitting or in sections at relevant timepoints. It is currently available as a physical A5 workbook and a pdf, with the potential to develop into an interactive website.

### Acceptability study

Qualitative interviews were conducted to explore the newly developed intervention’s acceptability with the target population.

i. *Participants*
  Study information was posted on relevant social media pages and websites to recruit a purposive sample of pregnant and postnatal UK women with a BMI ≥ 30 kg/m^2^ to participate in the study. Women were required to have had a BMI ≥ 30 kg/m^2^ at the start of their pregnancy and be either: at least 14 weeks pregnant and intending to breastfeed; or have birthed their baby and breastfed to any extent within the past 2 years.
ii. *Intervention review*
  Study information directed those interested in taking part to access a Participant Information Sheet, confirm their eligibility and complete a consent form via Qualtrics. The PIS included information on the team’s reasons for conducting the research, and expected outcomes (student dissertations, publication, future research). Upon receipt of consent form (*n* = 21), participants were contacted via email to share the intervention (pdf). The intervention was accessible on all digital devices. Participants were asked to read through the intervention before the interview, set at roughly one week after delivery.
iii. *Interviews*
  Interviews were conducted online, a method that is preferable to women with young children due to its convenience and accessibility [28]. Likewise, the method was appropriate for the analysis, as it allowed participants to remain relatively anonymous, and take part in the privacy of their own homes, which can facilitate the generation of rich data [29]. Thirteen telephone interviews were conducted between January and March 2024. Eight participants were unable to complete their interview at the time or reschedule. Information power considerations, such as the specific study aim and sample, the theoretically informed analysis and the richness of the data deemed this sample was sufficient to meaningfully address the research question [30].
  A semi-structured topic guide, reviewed by PPI group members (women with a BMI ≥ 30 kg/m^2^ who had breastfed) prior to conduction, was used. The TFAv2 was utilised to inform the interview schedule (Table 1). All interviews were conducted by female psychology student researchers (ET, RB, ZK) under supervision of SL. The student researchers had previous experience of conducting interviews with pregnant and postpartum women and were given training and support on qualitative interviewing prior to conduction by SL. Interviews were audio-recorded and transcribed verbatim, whilst removing any identifying information. Participants chose whether to select a pseudonym or allow the research team to generate one for them. Transcripts were not returned to participants for comment.
iv. *Data analysis*
  The data were analysed using hybrid thematic analysis (Table 2) [31]. A hybrid approach uses elements of both inductive and deductive coding to allow for an extensive and thorough analysis of the data in relation to a particular research question. Deductive coding allowed for the utilisation of the TFAv2, whilst inductive coding allowed identification of any other aspects of acceptability that were not captured within the framework. A realist approach to analysis was taken, to produce experiential themes that described their subjective viewpoints and experiences regarding the acceptability of the intervention [32]. Four researchers (SL, ET, RB & ZK) completed the coding process independently (steps 1–4), and then discussed and agreed themes (step 5). The themes and report were then reviewed by the full research team (step 6).

**Table 1:** Examples of interview questions informed by the Theoretical Framework of Acceptability v2 [16]

TFAv2 Construct	Example Interview Questions
**Affective attitude** *How an individual feels about an intervention*	• How did you feel about the breastfeeding workbook? • How did you feel about the images/quotes including women with higher BMIs?
**Burden** *The amount of effort that was required to participate in the intervention*	• How was the experience of accessing the workbook online? • How did you feel about the length/time it took to read the workbook and/or complete the activities?
**Ethicality** *The extent to which the intervention has a good fit with an individual’s value system*	• How did you feel about a breastfeeding workbook designed specifically for women with higher BMIs who want to breastfeed? • What, if any, might be the ethical issues with offering the workbook to women with higher BMIs?
**Intervention coherence** *The extent to which the participant understands the intervention and how it works*	• How did you find the introduction/glossary sections, which explained how the workbook was developed? • How understandable were these sections?
**Opportunity costs** *The extent to which benefits, profits or values must be given up to engage in the intervention*	• Can you think of anything that might make it difficult for other women to use the breastfeeding workbook? • What, if anything, might women with higher BMIs have to give up to receive/use the workbook?
**Perceived effectiveness** *The extent to which the intervention is perceived as likely to achieve its purpose*	• How far, if at all, do you think the workbook might help women with higher BMIs to breastfeed? • When do you think is the best time for women to receive the workbook?
**Self-efficacy** *The participant’s confidence that they can perform the behaviour(s) required to participate in the intervention*	• How understandable was the content of the workbook? • How did you find completing the activities?

**Table 2:** Steps of data analysis

Step of analysis	Description
1: Developing the coding manual	The TFAv2 and previous research was utilised to inform the initial coding manual
2: Testing the reliability of the code	Two transcripts were selected and coded using the manual by four researchers independently (ET, RB, ZK & SL). The researchers then met to discuss and make modifications to the manual accordingly
3: Summarizing data and identifying initial themes	To facilitate immersion in the data set, four researchers read all transcripts and produced bullet-point summaries of their content (ET, RB, ZK & SL). These summaries reflected the initial processing of the information by the researchers and provided the opportunity to begin to identify potential themes across participants
4: Applying template of codes and additional coding	Four researchers (ET, RB, ZK & SL) then coded all interview transcripts. Quotes were collated and organised under each code. Coding was guided, but not confined by the preliminary coding manual. Inductive codes were assigned to chunks of data that were relevant to the research question but did not fit with a preliminary code description. These inductive codes were added to the manual for all subsequent analysis of transcripts, and previously analysed transcripts were re-examined to identify any additional instances
5: Connecting the codes and identifying themes	Four researchers (ET, RB, ZK & SL) utilised the TFAv2 to connect semantically similar codes and identify themes across the data. Initial theme descriptions were written to facilitate step 6
6: Corroborating and legitimating coded themes	Quotes were closely scrutinized to ensure that themes and codes were grounded in the data. This step involved several iterations to develop solid theme descriptions. The final report was reviewed by all researchers

## Results

Thirteen interviews were conducted. Participants ranged in age (29–43 years, *Median* = 34), pre-pregnancy BMI (30–47 kg/m^2^, *Median* = 34.89) and ethnicity (9 White British, 4 Asian or Asian British). All participants had breastfed to some extent within the past two years, and their total breastfeeding experience ranged from 7 months to 8 years 10 months. One participant was currently pregnant and intending to breastfeed. Interview durations ranged from 27–58 min (*Median* = 37 min).

Data are presented in accordance with the TFAv2. TFAv2 constructs definitions which formed the framework for the analysis are presented in Table 1. All constructs featured in women’s experiences of the intervention and are reported in this analysis in order of prominence. Quotes are provided to illustrate themes.

### Perceived effectiveness

Participants believed the intervention would be effective in supporting breastfeeding throughout their journeys, from planning and developing realistic and achievable goals, to tackling and mastering latching and positioning during initiation, and maintaining breastfeeding, especially whilst in public and facing challenges such as pain or returning to work. This is because they believed the intervention promoted realistic expectations of breastfeeding; those who had breastfed felt the intervention captured their experiences accurately, whilst simultaneously providing additional useful knowledge.

*I think for me, all in all the workbook was-was brilliant because it's-it's the real truth. I mean, there's things that we do need to do, people should be aware of, for example, with the C-sections. No one really talks about breastfeeding after that, you know, the-how important skin to skin is (Chandra).*

Participants also highlighted the usefulness of the intervention acknowledging the challenges of breastfeeding and providing a wide variety of options to support them to overcome them. For example, the intervention prompts women to consider potential difficulties and problem-solve in advance, and includes information around alternatives to solely feeding from the breast, such as expressing (by hand or with a pump) and combination feeding, and seeking professional and social support.

*I think the sentence where it is, when when thinking about the cons of breastfeeding thinking of possible solutions in advance could reduce the negative impact. I think that's absolutely true. I think that's a good way to do it and to say, you know, what are your worries? Because yeah, you're right. If you've, if you've already kind of in your head gone, well this is my concern, if you've already got the comeback for it or a way of negating that feeling. I do think that's helpful. Yeah. (Sophie).*

Participants expressed the importance of including images and quotes from other women with higher BMIs who had breastfed. Feeling represented, and normalising breastfeeding in women with higher BMIs, especially beyond the first few months, was strongly valued, and linked to increasing breastfeeding duration.

*It's quite nice actually, having moms yeah, with real mums, real women actually participate in terms of that being in the images and having those quotes *etc. *And it's like, for instance, I think I noticed a lot of them in terms of when you're talking about the different positions yeah, so having a mom saying actually I tried this but this didn't work so then I was kind of guided to try and this in this work better this way for this child *etc. *So that was quite good (Kori).*

However, participants did acknowledge that the intervention may be less effective for those where breastfeeding is not the norm, and for those who have had negative encounters with others whilst breastfeeding. For example, women described experiencing guilt for breastfeeding against the advice of relatives, or for longer than is socially desirable, which could impact duration. The intervention encouraging support seeking, by signposting to breastfeeding helplines, websites and groups, may help women to overcome this barrier by changing their infant feeding environment.

*I noticed that the links, you know, it's got some of the important, like the National Breastfeeding Help line and, the Breastfeeding Network, you know, that you've got. I do think it's tricky to find your people and the people that can help. Because I think lots of people might go to the wrong…or down the wrong path if they don't know where to look. (Jessica, pg 5).*

For the intervention to be most effective, participants believed it should be delivered sensitively by healthcare professionals during mid-late pregnancy. Women believed introducing the intervention as a helpful resource to support their breastfeeding, rather than another intervention they were required to have because of their BMI, would be key to successful engagement. The intervention could also be delivered more widely, at children and family centres and breastfeeding groups.

*As early as possible, but I'd say not too early where you're kind of worried about scans an’ stuff. Once you know that baby's growing well doing well kind of so I'd say towards the end of the second or the third trimester (Anika).*

### Ethicality

Participants overwhelmingly agreed that it was ethical to design an intervention to support women with higher BMIs to breastfeed; women felt strongly that everyone deserved adequate breastfeeding support and felt positively that their needs were being addressed without inducing stigma or shame regarding their weight or infant feeding choices.

I was expecting because there can be a lot of sort of fat shaming around pregnancy and breastfeeding … and sometimes stuff that's aimed at ladies with higher BMI is very, it's supposed to be helpful but it comes across as quite, I don't know what the word is but it makes you feel s*** basically so I think I was really pleasantly surprised that there wasn't any of that in there (Dorothy).

Furthermore, the women agreed the intervention could bridge a gap in the support available by over-stretched health services; whilst it would not replace one-to-one professional or peer breastfeeding support, it could provide women with the information they needed during pregnancy, reducing time pressure in maternity appointments, and subsequently signposting them to helplines and local authority, charity or private groups and resources where National Health Service support is unavailable. Participants suggested adapting some images (e.g. of hand expressing) to illustrations, and including more information on how to breastfeed discreetly, could improve its acceptability for South Asian women.

*We had nursery nurses which was really nice because they came in they had a chat with me and they were there you know, any time they said just give us a call if you want help with breastfeeding we'll come along. But I personally would have preferred to have something like that [the intervention] to actually look at and leaf through and refer to (Elia).*

### Affective attitude

Participants reported liking the intervention’s interactive nature; they felt the activities were engaging, and providing example answers and prompts reduced the burden of completion. They also strongly appreciated feeling represented in the intervention (e.g. in the images and quotes) and being considered as a human with their own motivations and desires, beyond being purely a mother to an infant or a BMI status to be resolved. Including more images with greater diversity was recommended to increase this aspect further.

*I like the fact that it was interactive as such because you actually had sections where people had taken notes for themselves to answer questions *etc. *rather than just kind of being spoken out so to speak so I think those were quite nice for me (Kori).*

### Burden

Participants believed the time required to engage with the intervention was acceptable; they found the intervention easy to read and liked that they could access each section when convenient, rather than completing in one sitting. Times reported for completion ranged from 10 to 30 min, which was reduced with prior breastfeeding experience. Furthermore, some reported that the intervention reduced the time burden they associated with researching breastfeeding for themselves. Participants suggested burden could be further reduced by utilising summaries to condense more detailed sections and providing audio and video components to improve accessibility across a variety of situations and environments.

*Sometimes I think again that kind of postpartum period it's really really difficult… it's like well I can't be bothered to go through all of the research to try and find somewhere else, whereas you've got a nice big page of all these different things that you can go to in it it makes it a lot easier (Dorothy).*

### Intervention coherence

Participants reported understanding the purpose for the intervention and how it worked. The summary of the scientific underpinning was valued and was deemed to increase its legitimacy and likelihood of engagement. However, some participants did raise that labelling the intervention as ‘psychological’ may be less desirable to some women, when linked to their weight or breastfeeding. Therefore, careful language and explanations throughout are paramount to reduce this risk of avoidance.

*I think maybe the like saying the word psychological techniques might … might be off putting for some people so maybe like re-wording that might be helpful, because we have to acknowledge that when it comes to people's weight, is a very emotional kind of label attached to that as well, so as soon as you hear something about talking about the psychological elements of text related to somebody's weight for me that can be a little bit off-putting (Kori).*

### Self-efficacy

Participants reported feeling confident that they held the skills required to engage with the intervention; having ready access to a support network facilitated this further. However, providing translation options and alternatives to written text, such as audio and video content, was deemed key to equitable access and engagement.

*Yeah, language I can think of. If someone can't read properly, I don’t think writing is a bit of an issue or anything because I think as long as it makes you think it's fine. If you can't write it down, it's not a problem. But yeah, I'd say just language more than anything. Maybe if to help patients or you know, mothers who are unable to read then maybe more pictures. (Hena).*

### Opportunity costs

Participants reported the intervention had few opportunity costs. However, some acknowledged that the intervention may require distancing from some family or cultural traditions regarding infant feeding practices. For example, choosing to breastfeed may mean they are less available to fulfil other roles in the home, such as cleaning or taking care of other children. For those from families who traditionally formula feed, including information on being the first in the family to navigate how to breastfeed and being confident to make their own decisions for infant feeding was important. Furthermore, acknowledging that breastfeeding can have expenses, such as buying bras and other clothing, and pumps, sterilisers and bottles, was important to promote realistic expectations.

*I think from my three years of three years of being a mom I'd say recently there's people that want to breastfeed but before that, it's always been about bottle whatever’s the easy way out. We gotta get the cooking then we gotta do the cleaning. We gotta do this, we got this. So easy way out is a bottle because you can give the baby to anyone else and the baby kind of thing. Whereas breastfeeding is a very personal thing with mum and baby. And it's so important, but in an Asian culture, that's not what you think. (Hena).*

## Discussion

This study aimed to assess the acceptability of a breastfeeding intervention for women with a BMI ≥ 30 kg/m^2^. The TFA was applied to qualitative interviews exploring women’s experiences of receiving the intervention. The findings demonstrate that this intervention is acceptable to women with a BMI ≥ 30 kg/m^2^; the women liked the intervention, they felt that it was an appropriate method to support their breastfeeding behaviours, fit well within their value system and required a manageable amount of time and effort to complete. This increases the likelihood that the intervention would be delivered and completed as intended when trialled, increasing the accuracy of effectiveness evaluations [16].

Women with a BMI ≥ 30 kg/m^2^ believed the intervention would be effective at increasing breastfeeding initiation and duration. This could be explained by the intervention successfully targeting the psychological factors intended; women agreed that the intervention increased their positive beliefs, knowledge and confidence in their ability to breastfeed whilst promoting realistic expectations and avoiding inducing stigma or shame regarding their weight or infant feeding choices. The intervention also prompts the formation of detailed plans to breastfeed, and to seek a variety of professional and social support, which they believed would help them to overcome challenges. Behaviour change techniques were also successfully implemented, indicated by the women’s preference for and active participation with the interactive components. Separating the information into smaller chunks and including relatable images and quotes prompted engagement and improved the likelihood of its effectiveness [33–35].

Women believed the intervention could bridge a gap in current breastfeeding support. It is estimated that, globally, 900,000 more midwives are needed to improve maternity and postnatal care [36]; in the United Kingdom, health professionals often report not having enough time to provide quality breastfeeding support due to staffing shortages and short hospital stays [37, 38], and infant feeding leads report significant reductions in peer and specialist breastfeeding support groups (by 47% and 28% respectively), visits from health visitors (by 58%) and closure of children’s centres (by 48%) [39, 40]. Whilst women agreed the intervention could be acceptably delivered antenatally to provide them with the information they need during pregnancy, which could reduce pressures to discuss some aspects of infant feeding within maternity appointments, this should and could not replace professional and peer breastfeeding support; a key component to the likelihood of its success is its ability to effectively encourage and inform support seeking at the right time. Therefore, to ensure equitable access (and avoid reliance on private services), local authority and National Health Services should prevail to provide consistent specialised and peer breastfeeding support throughout the UK. It is also key that health professionals are supported to introduce the intervention sensitively, as perceived pressure to breastfeed or weight stigma would reduce engagement.

Further adaptations could improve the acceptability of the intervention. Women highlighted the importance of providing translation options, and alternatives to written text, such as audio and visual components. In the United Kingdom, adults of Black African, Black Caribbean, South Asian and White British ethnicities are the most likely to experience obesity [41]; the intervention could therefore be translated into the most common languages spoken amongst these groups, such as Punjabi, Urdu, Somali, Yoruba and French, to ensure equitable access [42]. Cultural adaptations, such as increasing confidence to break family feeding practices and traditions, and how to breastfeed in traditional or religious clothing, could also improve acceptability. Furthermore, globally, levels of health literacy, defined as “the ability of individuals to gain access to, understand and use information in ways which promote and maintain good health” [43] are negatively associated with increasing BMI [44]. Therefore, it is important that the intervention is presented as simply as possible; women in this study confirmed this, increasing its acceptability to the target population.

This study has several strengths. Conducting qualitative interviews allowed the in-depth exploration of women’s perceptions of the acceptability of the intervention, demonstrated by the median interview duration of 37 min. Applying the TFAv2 focused the analysis and structured the findings. Conducting the interviews online facilitated recruitment across the UK, and ease of participation for the target group (mothers with small children) [45]. The participants also varied in terms of their breastfeeding experience, pre-pregnancy BMI and ethnicity, broadening the views captured and likely improving the applicability of our findings to the target group.

This study also had limitations. Women volunteered to participate; individuals who self-select for health behaviour change interventions are more likely to seek social support and engage in active coping during stressful situations [46]. This may have influenced their perceptions of the intervention’s acceptability, as it encourages users to tackle breastfeeding challenges directly. However, active coping skills can be increased by engaging in problem solving [47], a skill that is also prompted and encouraged in the intervention. Furthermore, the current intervention and this study required women to read and understand English; future research should further explore the benefits of cultural adaptations and aim to include those for whom English is not their first language (~ 8% of UK population) [48].

Several implications are generated from this study. Findings suggest that the intervention is acceptable to women with a BMI ≥ 30 kg/m^2^, justifying the importance of progressing to the next phase of intervention development recommended in the MRC guidance [15], by conducting a pilot and randomised controlled trial to evaluate its effectiveness. As women identified the optimum delivery for the intervention as the perinatal period, an assessment of the intervention’s acceptability with health professionals delivering maternity care is also recommended. The findings suggest that targeting psychological factors associated with breastfeeding is also acceptable, which should be considered by health professionals delivering care; it is acceptable to discuss breastfeeding beliefs with women with a BMI ≥ 30 kg/m^2^, to help them to make realistic plans about breastfeeding, and to provide tailored information, without inducing stigma or shame regarding their weight or infant feeding choices.

## Conclusions

In conclusion, this study assessed the acceptability of a newly designed breastfeeding intervention for women with a BMI ≥ 30 kg/m^2^. The findings suggest that the intervention is acceptable and shows promise for increasing breastfeeding initiation and duration. The updated MRC guidance [15] offers a useful framework for developing complex interventions, and the TFAv2 provides structure and focus to explore acceptability. These findings can inform future research directions, intervention development, and maternity and breastfeeding care.

## Data Availability

The datasets used and analysed during the current study are available from the corresponding author on reasonable request.
